# Development and validation of the Disrespect and Mistreatment during Childbirth Questionnaire: risk factors and effects on parenting stress

**DOI:** 10.3389/fpsyg.2025.1562679

**Published:** 2025-03-05

**Authors:** Chiara Suttora, Odette Nardozza, Laura Menabò, Emanuele Preti, Ilenia Passaquindici, Mirco Fasolo, Maria Spinelli

**Affiliations:** ^1^Department of Psychology, “Renzo Canestrari”, University of Bologna, Bologna, Italy; ^2^Department of Psychology, University G. D'Annunzio Chieti-Pescara, Chieti, Italy; ^3^Department of Psychology, University of Milano-Bicocca, Milan, Italy

**Keywords:** childbirth experience, disrespect and abuse, obstetric mistreatment, birth trauma, parenting stress, measurement, factor analysis

## Abstract

**Introduction:**

A growing body of research indicates that disrespect and mistreatment during childbirth (obstetric mistreatment) are widespread globally. These experiences, along with their prevalence, risk factors, and impacts on maternal mental health, are often assessed using *ad hoc* tools, highlighting the need for psychometrically valid instruments. This study aims to develop and validate the Disrespect and Mistreatment during Childbirth Questionnaire (DMCQ) and explore factors contributing to negative childbirth experiences, as well as the relationship between mistreatment and parenting stress during the first two postpartum years.

**Methods:**

An online survey was administered to 620 women, assessing sociodemographic and childbirth-related factors, experiences of disrespect and mistreatment during childbirth, postpartum posttraumatic stress symptoms related to childbirth, personality traits, and parenting stress.

**Results:**

Exploratory factor analysis identified a 5-factor model with good internal consistency: negative interactions with healthcare providers, separation from the newborn, medical intrusiveness, verbal mistreatment, and pain experience. Confirmatory factor analysis supported this structure, showing positive correlations with perinatal stress (convergent validity) and no association with openness to experience (divergent validity). Higher scores on the DMCQ correlated with increased parenting stress, particularly distress related to the parental role. Women with higher education, low income, and births in Southern Italy reported greater obstetric mistreatment. Risk factors included primiparity, unplanned cesarean, instrumental delivery, episiotomy, anesthesia, labor exceeding 12 h, and delivery complications.

**Discussion:**

In conclusion, the Disrespect and Mistreatment during Childbirth Questionnaire is a psychometrically valid tool specifically designed to address obstetric mistreatment in the early years postpartum.

## 1 Introduction

Childbirth is a critical experience for women, with profound physical, psychological, social, and existential implications that extend both short-term and long-term (Stern and Bruschweiler-Stern, 1998). Beyond the medical dimensions of childbirth, a woman's subjective experience has been widely recognized as a crucial factor influencing her wellbeing and her bond with her baby. This perspective has become an increasingly prominent focus in recent research. In alignment with this growing interest, the present study seeks to validate a questionnaire designed to evaluate women's perceptions of childbirth, identify protective and risk factors, and explore their potential impact on the mother-child relationship.

The subjective perception of childbirth has a multidimensional nature as it depends on different factors such as antenatal risks, the physiological experience of labor and delivery, and its psychological evaluation (Harris and Ayers, 2012).

Among antenatal risks, the presence of pregnancy fears and anxiety can predict distress and complications during labor and delivery (Melender, 2002; Johnson et al., 2003). The negative appraisal of the current pregnancy, previous negative childbirth experiences, namely difficult or unplanned pregnancies, or a history of miscarriages, can also be determinant in this sense, leading to a traumatic childbirth experience (Armstrong, 2004; Edworthy et al., 2008). Moreover, factors unrelated to the experience of pregnancy or parenting can negatively affect the perception of childbirth. For instance, preexisting psychopathology, psychological trauma, and a history of sexual abuse are significant risk factors associated with maternal perception of childbirth as traumatic (Czarnocka and Slade, 2000; Soet et al., 2003).

The physiological and psychological aspects of the childbirth experience play a crucial role in determining maternal subjective perception of it. Such perceptions can depend on several factors, including the mode of delivery, the duration of labor and delivery, the use of medications such as anesthesia, and the occurrence of emergencies during childbirth. Different delivery methods (i.e., spontaneous vaginal, instrumental vaginal, or planned and unplanned cesarean section) are associated with various maternal perceptions of childbirth (Velho et al., 2012; Carquillat et al., 2016). The choice of instrumental or cesarean delivery usually involves high-risk or emergency situations that can pose threats to maternal and infant health and safety. Instrumental vaginal delivery, including forceps- or vacuum-assisted procedures—as well as other gynecological procedures (i.e., episiotomy) can lead to peri- and post-partum medical complications that can contribute to the development of adverse mental health outcomes, as stress, somatization, obsessive-compulsive symptoms, depression, and anxiety (Dekel et al., 2019; Djanogly et al., 2022). Furthermore, cesarean sections—whose rates have significantly increased in the last decades, reaching percentages around 21%, surpassing the ideal acceptable rate of 10–15% according to the WHO (Angolile et al., 2023)— expose mothers and infants to short and long-term complications, increasing the chances of negative outcomes.

The physiological and psychological aspects of childbirth are deeply interconnected. Particularly, the perceived quality of interaction with healthcare providers often plays a pivotal role in shaping a woman's childbirth experience. Feeling informed about medical procedures and decisions, supported, and cared for during labor and delivery can profoundly enhance the childbirth experience. Conversely, feeling ignored, unsupported, or abandoned by medical staff can significantly worsen maternal perceptions of childbirth. In recent decades, terms such as obstetric mistreatment or obstetric violence (for a discussion on terminological nuances, see Chervenak et al., 2024; Vullikanti and Yamin, 2024) have been introduced to describe various forms of abuse within obstetric care. In the present study, we decided to refer to the phenomenon using the term *obstetric mistreatment* to emphasize the perception of inadequacy in the obstetric care received by the woman. Bohren et al. (2015) provided an evidence-based classification of mistreatment during childbirth in health facilities based on a review of 65 studies. This classification includes physical abuse, sexual abuse, verbal abuse, stigma and discrimination, failure to meet professional standards of care, poor rapport between women and providers, and issues with health facilities. A subsequent review by Darilek (2018) identified four primary categories of obstetric mistreatment: physical abuse (e.g., rough or unnecessary vaginal examinations and episiotomies); verbal abuse (e.g., scolding, verbal threats); overmedicalization of childbirth (e.g., lack of informed consent, coercion, and unnecessary interventions); and neglect, lack of dignity, and discrimination (e.g., withholding care or showing disrespect toward the woman). Research on the prevalence of obstetric mistreatment revealed that the phenomenon is alarmingly prevalent even within well-resourced healthcare systems (Martínez-Galiano et al., 2021; van der Pijl et al., 2022; Garcia, 2023). In response to this widespread issue, the World Health Organization (WHO) has identified disrespect and abuse during childbirth in medical facilities as a priority for assessment, prevention, and eradication (World Health Organization, 2014).

Focusing on European contexts, Martínez-Galiano et al. (2021) conducted a study in Spain investigating three components of obstetric mistreatment: physical, verbal, and psycho-affective. The research was based on an online survey completed by 899 women who had given birth in the previous 12 months. The psycho-affective dimension included preventing the presence of a support person during labor, restricting contact with the newborn after birth, and feelings of lack of collaboration, vulnerability, guilt, and insecurity imposed on the woman. Two out of three women, 67.4% of the sample, reported at least one experience of obstetric mistreatment. van der Pijl et al. (2022) investigated disrespect and abuse during labor and birth in the Netherlands from a large sample of 12,239 women. In an online survey, women reported various forms of mistreatment, including lack of communication and support and harsh and rough treatment or physical violence. One out of three women—especially primiparous and migrant women—considered these experiences as upsetting, reporting a negative birth experience. In Italy, both Ravaldi et al. (2018) and Scandurra et al. (2022) reported data about obstetric mistreatment, its prevalence, associated risk factors, and related issues. The first (Ravaldi et al., 2018) conducted an online interview with women with children aged 0 to 14 years. Among them, 21% identified themselves as victims of obstetric mistreatment, as defined in the study. This included the appropriation of their bodies by healthcare providers, unnecessary obstetric procedures, excessive exposure of nudity, separation from their newborns, exclusion from medical decision-making, and instances of verbal humiliation. Additionally, 33% of participants reported feeling inadequately supported during their obstetric care. The study reported that rates of obstetric mistreatment were higher in the central and southern regions of Italy. More recently, Scandurra et al. (2022) investigated the types and prevalence of obstetric mistreatment in a sample of 282 women aged 18 to 60 using a translated version of the questionnaire developed by Castro and Frías (2019) in Mexico aimed to assess two factors: abuse and violence, and non-consented care. Consistent with findings from Spain and the Netherlands, over 75% of participants reported experiencing at least one form of obstetric mistreatment. Younger women and those with lower levels of education reported higher rates of obstetric mistreatment, while women who attended prenatal childbirth courses or had a vaginal delivery reported lower rates. The authors also provided valuable insights on the impact of obstetric mistreatment on maternal mental health: women scoring higher on the abuse and violence factor dimension also reported greater psychological distress and symptoms of post-traumatic stress. These findings align with a growing body of literature documenting the mental health consequences of obstetric mistreatment (Martinez-Vázquez et al., 2022; Silva-Fernandez et al., 2023). For example, Martinez-Vázquez et al. (2022) in Spain, Silveira et al. (2019), and de Souza et al. (2017) in Brazil observed that both verbal and psycho-affective obstetric violence represented a relevant risk factor for the development of postpartum depression within a year after childbirth. Through a systematic review of 21 articles, Silva-Fernandez et al. (2023) explored the medical and psychological factors associated with obstetric mistreatment and their impact on women's mental health outcomes, specifically postpartum depression (PPD) and post-traumatic stress disorder (PTSD). The mode of delivery, when instrumental or cesarean, constituted a significant risk factor for both PPD and PTSD, whereas specific obstetric interventions such as several perineal tears, the Kristeller technique, and labor induction were identified as risk factors uniquely for PTSD (see among others Hernández-Martínez et al., 2019; Martinez-Vázquez et al., 2021). Importantly, partner support during labor and high satisfaction with healthcare services during birth were identified as protective factors for PPD. Similarly, respect for the labor plan, adequate communication with healthcare professionals, social support during labor, and the skin-to-skin procedure were protective factors for PTSD. These findings emphasize the critical role of respectful and supportive care during childbirth in mitigating the psychological impact of obstetric experiences. Given the significant repercussions of obstetric mistreatment on women's mental health and the potential long-term consequences for the mother-infant relationship, addressing these issues demands immediate and urgent attention.

### 1.1 The current study

Given the widespread occurrence of the phenomenon, even in medium- and high-income countries, this study seeks to contribute to the understanding of obstetric mistreatment by addressing three main objectives. Because most of the previous investigations on obstetric mistreatment assessed it focusing only on the medical procedures associated with it, the first aim of this study is to develop and validate a questionnaire specifically designed to assess the subjective perception of obstetric mistreatment and its dimensions. Moreover, while previous studies, as evidenced in the review by Silva-Fernandez et al. (2023), often relied on *ad hoc* tools or validated instruments designed to measure positive childbirth experiences (e.g., the Birth Satisfaction Scale; Hollins Martin and Martin, 2014), this study introduces the Disrespect and Mistreatment during Childbirth Questionnaire (DMCQ). The DMCQ aims to capture the subjective experience of disrespect and mistreatment during labor and childbirth, emphasizing the negative aspects often overlooked in existing measures. Based on a comprehensive literature review, the hypothesized dimensions of obstetric mistreatment included: perceived control during childbirth; physical, verbal, and psychological maltreatment or abuse; lack of consent on obstetric procedures; excessive medicalization; pain management therapy; separation from the newborn; quality of the interactions with healthcare providers. To address this aim (aim 1), data will be collected from mothers with children aged 0 to 2 years to examine: (a) the factorial structure of the DMCQ and its dimensions and internal consistency and (b) the questionnaire's convergent and divergent validity.

The second aim (aim 2) of the study is to investigate the individual and contextual factors potentially contributing to negative childbirth experiences associated with obstetric mistreatment. We will consider sociodemographic and childbirth-related factors.

While previous research has documented associations between obstetric mistreatment and adverse maternal mental health outcomes (i.e., post-traumatic stress disorder and postpartum depression), little is known about the impact of obstetric mistreatment on the early mother-child relationship. Therefore, this study aims (aim 3) to bridge this gap by examining the influence of negative childbirth experiences on parenting stress during the first 2 years postpartum.

Additionally, our fourth aim (aim 4) focuses on establishing a clinical cut-off for the questionnaire to identify women who experience higher levels of disrespect and mistreatment and are, therefore, at greater risk of adverse outcomes, such as perinatal and parenting stress. Moreover, this aim seeks to characterize high-risk women by analyzing the impact of individual and contextual risk factors on their likelihood of experiencing disrespect and mistreatment during childbirth.

The decision to focus on mothers of children aged 0 to 2 years ensures the collection of insights that are both timely and directly relevant to obstetric mistreatment—a phenomenon increasingly recognized due to global attention and ongoing shifts in the medicalization of childbirth. In contrast with previous studies in Italy (Ravaldi et al., 2018; Scandurra et al., 2022), which comprised mothers of children across a wide age range, including adolescents, this narrower sampling approach mitigates methodological challenges. By concentrating on mothers with recent childbirth experiences, the study ensures a more precise examination of how obstetric mistreatment impacts maternal mental health and early parenting dynamics.

## 2 Methods

### 2.1 Participants and procedures

Participants were *N* = 620 mothers (*M*_*age*_ = 35.41 years, *SD* = 4.61) who completed an online survey 0 to 24 months postpartum. The survey assessed sociodemographic and childbirth-related factors, experiences of disrespect and mistreatment during childbirth, postpartum posttraumatic stress symptoms related to childbirth, personality traits, and parenting stress. The exclusion criteria for the study were refusal to participate by not giving consent and being under 18 years of age. Participants were recruited through social media, with the survey created on the Qualtrics platform and shared via an anonymous online link. Participants gave their consent to participate by clicking on the consent box before answering the survey, which included a detailed study description and ethical considerations. Participation was voluntary and not remunerated. The sample's characteristics are illustrated in Table 1.

**Table 1:** Sociodemographic and clinical characteristics of the sample (*N* = 620).

**Variables**	**N**	**M (SD)/n (%)**
**Sociodemographic information**		
Maternal age	620	35.41 (4.61)
Maternal education	620	
*Elementary/High school (1)*		236 (38.1)
*University/Postgraduate degree (2)*		384 (61.9)
Mother Italian nationality	620	603 (97.3)
Socioeconomic status/monthly income	594	
*Less than 2150€*		222 (35.8)
*More than 2150€*		372 (60.0)
Marital status during pregnancy	620	
*Married/Cohabitating/in a relationship*		613 (98.9)
Hospital Area in Italy	620	
*South (1)*		212(34.2)
*Center (2)*		77 (12.4)
*North (3)*		331 (53.4)
Child sex (F)	620	305 (49.2)
Child age	620	13.63 (5.01)
Baby born full term	620	566 (91.3)
Firstborn child	620	495 (79.8)
**Childbirth related factors**		
Type of delivery	620	
*Vaginal delivery*		405 (65.3)
*Instrumental delivery*		48 (7.7)
*Cesarean section (scheduled)*		56 (9)
*Cesarean section (emergency)*		111(17.9)
Duration of labor (>12 h)	561	190 (30.6)
Episiotomy	607	101 (16.3)
Anesthesia	619	193 (38)
Complications during childbirth	610	61 (9.8)
Newborn's health complications	569	123 (19.8)

Italicized words represent the levels of categorical variables.

### 2.2 Measures

#### 2.2.1 Sociodemographic information

A self-report questionnaire was used to record the following information: maternal age, maternal education level, nationality, socioeconomic status, and marital status during pregnancy. Information about the hospital area was also collected to examine differences in childbirth experiences among women from northern, central, and southern Italy. Child-related information was collected through questions about the child's gender, age, preterm childbirth, and birth order (e.g., firstborn).

#### 2.2.2 Childbirth-related factors

Regarding childbirth-related factors, mothers provided information about the type of delivery (e.g., vaginal birth, instrumental delivery, scheduled cesarean section, or emergency cesarean section), the duration of labor (i.e., whether it lasted more or less than 12 hours), the use of medical interventions (e.g., episiotomy or anesthesia), and any complications during birth, including those affecting the newborn's health.

#### 2.2.3 Item pool for the disrespect and mistreatment during childbirth questionnaire

Based on the growing literature on childbirth experience presented in the introduction section, two of the authors (CS and EP)—psychologists with expertise in developmental and clinical psychology, as well as in the field of perinatal care—generated an initial pool of 40 items. in generating the items, the recommendations of Hinkin (1998) were followed, avoiding double-barreled items and leading questions, using a limited number of reverse-scored items, and keeping the items as simple and short as possible. based on the literature reviewed in the introduction, we identified areas of particular interest for the topic and used those areas as guidelines for item generation: (1) physical, verbal, or psychological mistreatment (e.g., I was insulted; During childbirth, I received demeaning remarks); (2) perceived control during childbirth (e.g., I had an active role in the childbirth); (3) lack of consent on obstetric procedures and excessive medicalization (e.g., I felt subjected to unnecessary procedures; I underwent obstetric procedures without prior notice); (4) quality of the interactions with healthcare providers and perceived support (e.g., The staff was always available); (5) separation from the newborn (e.g., After delivery, I was not as close to my baby as I would have liked); (6) pain management (e.g., I experienced more pain than I expected; The medical staff delayed too long in providing me with pain relief). This initial set of 40 items was piloted with a small sample of mothers who had recently given birth as part of two psychology master's thesis projects at the University of Milano-Bicocca. Participants were asked to indicate how much each statement was descriptive of their childbirth experience on a seven-point likert scale ranging from 1 “not at all” to 7 “very much”.

The pilot resulted in the exclusion of 10 items due to inconsistent scores and concerns that they were overly wordy or difficult to understand (e.g., “The timely information provided by the medical and healthcare staff has reduced my concerns about my health and that of the baby,” and “I was always explained why it was necessary to undergo a certain procedure, even though I was opposed to it”). As a result, the final item list consisted of 30 items.

#### 2.2.4 Perinatal PTSD questionnaire (PPQ-II)

The PPQ-II is a 14-item questionnaire that assesses childbirth-related posttraumatic symptoms (Callahan and Borja, 2008). In the present study, we applied the recent Italian validation of the instrument (Nardozza et al., 2025) composed of 10 items. the global index of general PTSD symptoms associated with childbirth was computed (Cronbach's α = 0.82). Mothers were asked to indicate on a five-point likert scale (0 = not at all to 4 = often, for more than a month) how often they experienced the symptoms after childbirth.

#### 2.2.5 Personality traits

To investigate the divergent validity of the DMCQ we used the Italian version of the Big Five Inventory - 10 (BFI-10; Guido et al., 2015) to assess personality traits according to the five-factor approach (McCrae and Costa, 1994). It is composed of 10 items aimed at measuring five dimensions of personality. For the current study, we considered only the two items of the openness to experience scale (e.g., “I see myself as someone who has an active imagination”), reflecting the degree of intellectual curiosity, creativity, and a preference for novelty and variety. Each item is rated on a five-point Likert scale ranging from 1 = Strongly disagree to 5 = Strongly agree. The sperman-brown coefficient in the current study was 0.47, similar to the 0.50 of the validation study (Guido et al., 2015).

#### 2.2.6 Parenting stress index—short form

The parenting stress index—short form (PSI-SF; Abidin et al., 1997; Italian validation by Guarino et al., 2008) is a commonly used questionnaire designed to measure stress in the parent-child system and to identify those caregivers who are most in need of support. The PSI-SF includes 36 items rated on a five-point Likert scale (1 = strongly disagree; 5 = strongly agree) and consists of three subscales, each including 12 items: parental distress (PD), parent-child dysfunctional interaction (P-CDI), and difficult child (DC). High values indicate more parenting stress. For the purpose of this study only the parental distress and the parent-child dysfunctional interaction were included (Cronbach's α: PSI PD = 0.81; PSI P-CDI = 0.72). The parental distress subscale explores the stress related to the parent's perception of her/his child-rearing competences, the level of spousal conflicts or support, and the restrictions placed by parental role. The parent-child dysfunctional interaction subscale refers to the parent's perception of difficulties in the parent-infant interaction.

### 2.3 Plan of analysis

Considering our first aim, the development and validation of the Disrespect and Mistreatment during Childbirth Questionnaire, we examined the factor structure of the DMCQ through exploratory factor analysis (EFA) using parallel analysis and Promax rotation and, following a confirmatory factor analysis (CFA). Prior to conducting the analyses, we pseudo-randomly divided the sample into two halves while controlling for child age and birth order. The first half was used to identify the underlying factor structure through exploratory factor analysis (EFA), and the second half was used to confirm this structure via confirmatory factor analysis (CFA). For all subsequent analyses, the entire sample was used. We also performed reverse coding on the following items to align with the theoretical direction of our scales: items 4, 5, 9, 10, 13, 15, 16, 20, 21, 22, 25, and 27.

As for the EFA, the sample's suitability for factor analysis was evaluated using the Kaiser-Meyer-Olkin (KMO) measure and Bartlett's test of sphericity. We computed the Marker Index (MI; (Gallucci and Perugini, 2007) to differentiate primary and secondary loadings, reducing cross-loadings and ensuring a clearer factor solution. Items with MI values above 0.40 were considered strong indicators of their respective factors, while those below this threshold were removed.

Next, a CFA was performed to test the adequacy of the factor structure identified with EFA. Given the sensitivity of the chi-square index to sample size, model fit was evaluated using four additional indicators: Comparative Fit Index (CFI), Root Mean Square Error of Approximation (RMSEA), Goodness of Fit Index (GFI), and Standardized Root Mean Square Residual (SRMR). Following established criteria (Browne and Cudeck, 1992; Hu and Bentler, 1999), acceptable model fit was indicated by CFI values ≥ 0.90, RMSEA ≤ 0.08, and SRMR ≤ 0.10. We also reported the relative chi-square (χ^2^/df), with values below 5 indicating good or acceptable fit (Arbuckle, 2011). We assessed internal consistency using Cronbach's alpha coefficients for each subscale and the overall scale. Item-total correlations were calculated, with values above 0.30 deemed acceptable (Moreira and Canavarro, 2017). In addition, convergent and divergent validity was examined on the entire sample by performing Pearson's correlations between the DMCQ total scale and the Italian Version of the Modified Perinatal Post-Traumatic Stress Disorder Questionnaire (PPQ-II) and the Big Five Openness scale, respectively.

To address Aim 2, which involved identifying individual and contextual risk factors associated with negative childbirth experiences, *t*-tests and ANOVA were employed. For Aim 3, which focused on examining the impact of negative childbirth experiences on parenting stress, a series of Pearson correlation analyses were performed. Regarding Aim 4, the clinical cut-off for the DMC questionnaire was determined by calculating the 90th percentile. Women scoring below and above this threshold were compared using *t*-tests to assess their perinatal stress (PPQ) and parenting stress levels. Additionally, individual and contextual risk factors were analyzed for women above and below this cut-off using *t*-tests and ANOVA. All analyses were performed using SPSS version 28 and R software, employing the Paran package for parallel analysis, the Psych package for EFA, and the Lavaan package for CFA using maximum likelihood estimation (ML).

## 3 Results

### 3.1 Development and validation of the disrespect and mistreatment during childbirth questionnaire (aim 1)

#### 3.1.1 Exploratory factor analysis (sample 1)

The Kaiser-Meyer-Olkin (KMO) test (KMO = 0.89) and Bartlett's test of sphericity [χ^2^ = 6429.719, df = 435, *p* < 0.001] confirmed that the sample was suitable for factor analysis. Parallel analysis initially identified six eigenvalues >1. However, the marker index revealed that items 3, 7, 15, 18, 20, 21, 22, and 29 had loadings below 0.40, leading to their removal. With 22 items remaining, a second parallel analysis was conducted, which indicated five eigenvalues >1. Despite this, the marker index showed that items 4 and 9 were still inadequate. Consequently, the final structure of the scale consisted of 20 items grouped into five factors (see Table 2).

**Table 2:** Factor loadings after principal axis factoring with promax rotation.

**Items**	**DMCQ NI**	**DMCQ SN**	**DMCQ MI**	**DMCQ VI**	**DMCQ PE**
5. The staff was always available.	**0.94**	−0.03	−0.06	0.05	−0.04
10. I received the right support from the healthcare staff.	**0.89**	−0.05	0.06	−0.07	−0.04
13. I always knew whom to turn to in case of need.	**0.84**	0.02	0.05	−0.06	0.00
27. The healthcare staff understood my needs.	**0.78**	0.04	−0.01	0.06	0.06
2. I felt abruptly separated from my baby.	0.00	**0.79**	0.07	0.07	−0.07
16. My baby was as close to me as I desired.	0.05	**0.87**	−0.06	−0.05	0.05
23. After giving birth, I was not as close to my baby as I would have liked.	−0.07	**0.58**	0.05	−0.07	0.10
26. I wished for greater contact with my newborn.	−0.02	**0.82**	−0.02	−0.02	−0.05
30. I could not see my baby for what I considered an excessive period of time.	0.04	**0.77**	−0.05	0.12	−0.09
1. I felt subjected to unnecessary procedures.	0.07	0.03	**0.65**	0.04	0.14
8. I don't know why certain procedures were performed.	0.07	0.07	**0.66**	0.05	0.02
14. I found some obstetric procedures I underwent excessively invasive.	0.03	−0.03	**0.71**	0.12	0.02
19. I underwent obstetric procedures without prior notice.	−0.06	0.03	**0.86**	−0.12	−0.10
28. I did not give consent for some procedures I received.	0.00	−0.05	**0.66**	−0.01	−0.03
6. The healthcare staff used vulgar language.	0.04	0.05	−0.15	**0.86**	−0.04
12. I was insulted.	−0.06	0.01	0.04	**0.88**	−0.03
17. During childbirth, I received demeaning remarks.	−0.01	−0.06	0.11	**0.64**	0.07
11. I experienced more pain than I expected.	−0.05	−0.05	0.04	−0.03	**0.76**
24. I feared I wouldn't make it because of the intense pain.	−0.06	−0.06	−0.07	−0.01	**0.88**
25. I felt capable of facing the experience.	0.10	0.12	−0.03	0.01	**0.64**
**Items removed with Marker Index** ** < 0.40**					
3. The healthcare staff delayed too long in providing me with pain relief.					
4. They made me feel like a bad parent.					
7. I was able to choose the partner I wanted to accompany me during labor and delivery.					
9. I felt judged as a mother.					
15. I had an active role in the childbirth.					
18. Even though I needed it, I was not provided with adequate pain relief.					
20. I had control over choosing the position I preferred during childbirth.					
21. The healthcare staff informed me about all labor and childbirth stages.					
22. The way the delivery took place was the one I desired.					

DMCQ NI, negative interactions with healthcare providers; DMCQ SN, separation from the newborn; DMCQ MI, medical intrusiveness; DMCQ VI, verbal mistreatment; DMCQ PE, pain experience. Bolded items represent factor loadings that indicate saturation on the respective dimension.

The following factors and corresponding subscale names were identified for the Disrespect and Mistreatment during Childbirth Questionnaire. The first factor, including items 5, 10, 13, and 27, was defined “Negative interactions with healthcare providers” (NI). Items saturating this factor regard the availability of healthcare staff, the support received, and their ability to understand the patient's needs. The second factor, “Separation from the newborn” (SN), included items 2, 16, 23, 26, and 30. These statements reflect feelings of being suddenly and inexplicably separated from the newborn, as well as the desire for greater support in fostering contact with the baby. The third factor, “Medical intrusiveness” (MI) (items 1, 8, 14, 19, and 28), pertains to experiences of undergoing medical procedures without proper consent or explanation or procedures perceived as invasive or unnecessary. The fourth factor, “Verbal mistreatment” (VM) (items 6, 12, and 17), encompasses experiences of being insulted, subjected to negative remarks, or addressed with vulgar language. The last factor, called “Pain experience” (PE) (items 11, 24, and 25), regards experiencing too much or unbearable pain during childbirth. Overall, the model accounted for 62% of the total variance. Specifically, Negative interactions with healthcare providers contributed 15% to the explained variance, while Separation from the Newborn also explained 15%. Additionally, Medical Intrusiveness accounted for 13%, Verbal Mistreatment for 10%, and Pain Experience for 9% of the variance.

#### 3.1.2 Confirmatory factor analysis (sample 2)

**Figure 1:**
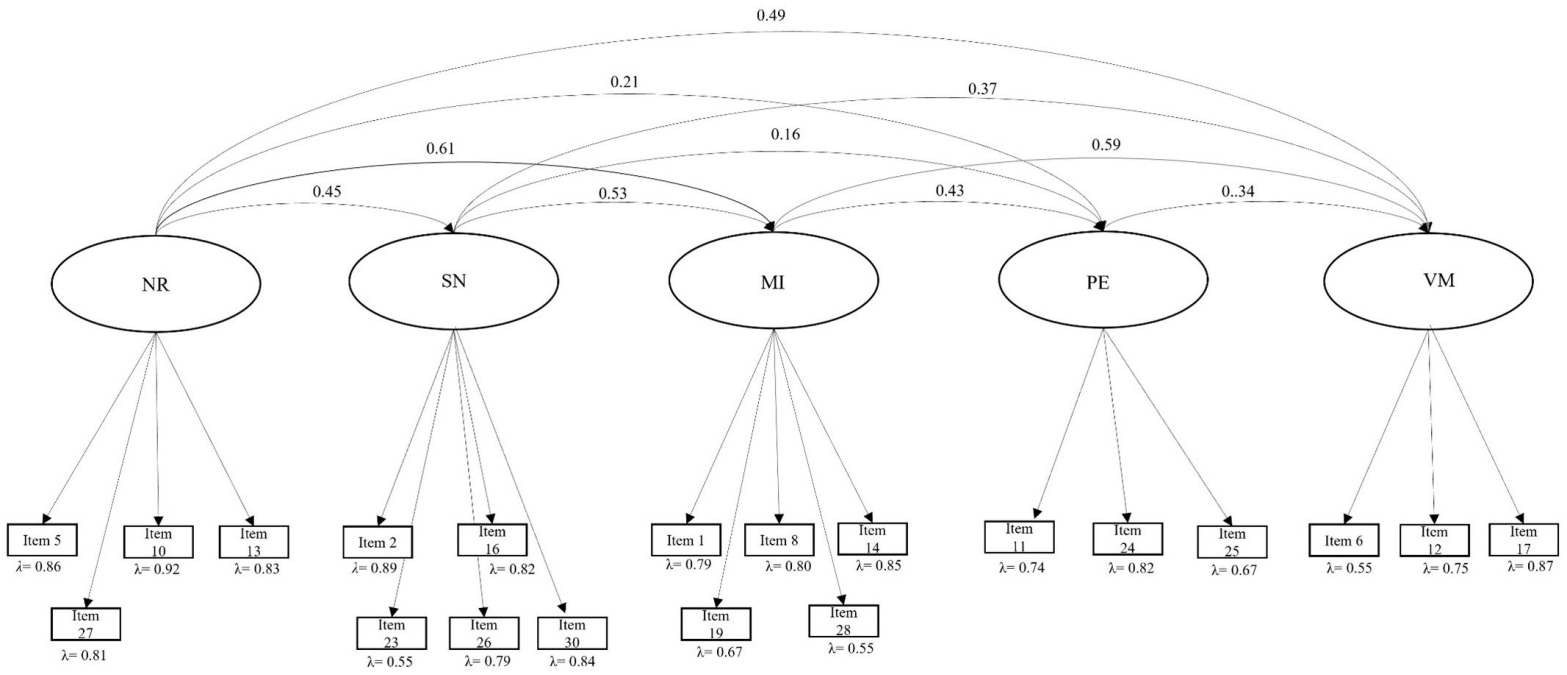
Five-factor structure of the disrespect and mistreatment during childbirth questionnaire. NI, negative interaction with healthcare providers; SN, separation from the newborn; MI, medical intrusiveness; VI, verbal mistreatment; PE, pain experience.

The correlated five-factor model presented an acceptable fit to the data, χ^2^(160) = 462.14, *p* < 0.001; χ^2^/df = 2.66; CFI = 0.93; RMSEA = 0.07; SRMR = 0.07, TLI = 0.92. These fit scores indicate that the model adequately represents the data. All the standardized factor loadings were significant (*p* < 0.001), ranging from 0.54 (item 28) to 0.92 (item 10) (Figure 1). This indicates that all items significantly contribute to their respective factors, with factor loadings reflecting the strength of these contributions. specifically, loadings for each factor were as follows: factor 1 (negative interactions with healthcare providers) loadings ranged from 0.81 (item 29) to 0.92 (item 10); factor 2 (separation from the newborn) loadings ranged from 0.55 (item 23) to 0.88 (item 2); factor 3 (medical intrusiveness) loadings ranged from 0.54 (item 28) to 0.85 (item 14); factor 4 (verbal mistreatment) loadings ranged from 0.55 (item 6) to 0.87 (item 17); and factor 5 (pain experience) loadings ranged from 0.67 (item 25) to 0.82 (item 24). The highest correlations were observed between factor 1 (negative interactions with healthcare providers) and factor 3 (medical intrusiveness) (ϕ = 0.613, *p* < 0.001) and factor 3 (medical intrusiveness) and factor 4 (verbal mistreatment) (ϕ = 0.590, *p* < 0.001), indicating a strong relationship between these constructs. The weakest covariances were found between factor 2 (separation from the newborn) and factor 5 (pain experience) (ϕ = 0.160, *p* = 0.049) and factor 1 (negative interactions with healthcare providers) and factor 5 (pain experience) (ϕ = 0.209, *p* = 0.017), suggesting a weaker relationship between these latent dimensions.

#### 3.1.3 Reliability analysis and convergent validity

Good Cronbach's alpha values were found for all the factors: negative interaction with healthcare providers (α = 0.92), separation from the newborn (α = 0.88), medical intrusiveness (α = 0.85), verbal mistreatment (α = 0.79) and pain experience (α = 0.78). The Cronbach's alpha for the total scale was 0.90.

As shown in Table 3, all corrected item-total correlations were above 0.30, with the exception of item 24, “I feared I wouldn't make it because of the intense pain,” from factor 5 (Pain experience), which showed a correlation of 0.30. Finally, as presented in Table 4, all subscales were significantly and strongly correlated with the total score, and all correlations between subscales were significant. Convergent and divergent validity were confirmed by examining the associations between the Disrespect and Mistreatment during Childbirth (DMCQ), perinatal PTSD (PPQ-II), and the Big Five openness personality trait (BFopen). As shown in Table 5, the DMCQ total score exhibited a positive and significant correlation with the PPQ-II, while the correlation with the BF openness score was non-significant.

**Table 3:** Mean scores, standard deviation and range for the Items and Reliability Analysis for each item.

	***N***	***Mean (SD)***	**Range**	**Corrected item-total correlation**	**Cronbach alpha if items deleted**
**Negative interaction with healthcare providers**					
Item 5	620	3.08 (1.88)	1–7	0.66	0.89
Item 10	620	3.11 (1.89)	1–7	0.69	0.89
Item 13	620	3.45 (1.97)	1–7	0.65	0.89
Item 27	620	3.14 (1.81)	1–7	0.75	0.89
**Separation from the newborn**					
Item 2	620	2.30 (2.01)	1–7	0.66	0.89
Item 16	620	2.61 (2.11)	1–7	0.59	0.90
Item 23	620	2.72 (2.78)	1–7	0.39	0.90
Item 26	620	3.49 (2.56)	1–7	0.54	0.90
Item 30	620	2.08 (1.87)	1–7	0.59	0.90
**Medical intrusiveness**					
Item 1	620	2.22 (1.81)	1–7	0.71	0.89
Item 8	620	2.28 (1.90)	1–7	0.70	0.89
Item 14	620	2.19 (1.82)	1–7	0.71	0.89
Item 19	620	1.73 (1.59)	1–7	0.57	0.90
Item 28	620	1.68 (1.53)	1–7	0.48	0.90
**Verbal mistreatment**					
Item 6	620	1.36 (1.06)	1–7	0.43	0.90
Item 12	620	1.32 (1.02)	1–7	0.55	0.90
Item 17	620	1.31 (1.06)	1–7	0.57	0.90
**Pain experience**					
Item 11	620	3.88 (2.27)	1–7	0.33	0.90
Item 24	620	3.80 (2.22)	1–7	0.30	0.90
Item 25	620	3.30 (1.72)	1–7	0.51	0.90

**Table 4:** Descriptive statistics for disrespect and mistreatment during childbirth questionnaire (DMCQ) total and subscales scores and correlations between the DMCQ subscales and total score.

	**N**	**M**	**SD**	**1**	**2**	**3**	**4**	**5**	**6**
1. DMCQ Tot	620	51.07	21.39	–					
2. DMCQ NI	620	12.78	6.77	0.76^***^	–				
3. DMCQ SN	620	13.20	8.89	0.73^***^	0.38^***^	–			
4. DMCQ MI	620	10.11	6.90	0.81^***^	0.56^***^	0.41^***^	–		
5. DMCQ VM	620	3.99	2.64	0.57^***^	0.42^***^	0.29^***^	0.44^***^	–	
6. DMCQ PE	620	10.98	5.21	0.50^***^	0.21^***^	0.12^**^	0.34^***^	0.22^***^	–

DMCQ Tot, disrespect and mistreatment during childbirth total score; DMCQ NI, negative interaction with healthcare providers; DMCQ SN, separation from the newborn; DMCQ MI, medical intrusiveness; DMCQ VM, verbal mistreatment; DMCQ PE, pain experience. ^**^p < 0.01, ^***^p < 0.001.

**Table 5:** Convergent and divergent validity: correlations between disrespect and mistreatment during childbirth questionnaire (DMCQ), PPQ-II total score (convergent), and big five openness score (divergent).

	***N***	***M***	***SD***	**1**	**2**
1. DMCQ Tot	620	51.07	21.39	–	
2. PPQ-II	620	2.41	0.80	0.56^***^	–
3. BFopen	575	4.14	1.04	−0.02	−0.09^*^

DMCQ Tot, disrespect and mistreatment during childbirth total score; PPQ-II Tot, modified perinatal post traumatic stress disorder questionnaire total score; BFopen, big five openness score. ^*^p < 0.05. ^***^p < 0.001.

The validated version of Italian Disrespect and Mistreatment during Childbirth Questionnaire is provided in Table S1 in the Supplementary material.

### 3.2 Identifying individual and contextual factors associated with the experience of disrespect and mistreatment during childbirth (aim 2)

Associations between the total score of the Disrespect and Mistreatment during Childbirth Questionnaire and various individual and contextual variables were examined, including women's sociodemographic status and childbirth-related characteristics. Neither child age, *r* = 0.06, *p* = 0.13, nor maternal age, *r* = 0.02, *p* = 0.64, at the time of survey administration, showed significant associations with women's scores on the questionnaire. Mothers with higher levels of education reported more negative childbirth experiences (*M* = 52.52, *SD* =22.43) than mothers with lower educational levels (*M* =48.71, *SD* =19.40), *t*_(551.37)_ = −2.24, *p* =0.026. Furthermore, differences emerged based on monthly income, with women of lower socioeconomic status (*M* = 53.97, *SD* = 21.62) reporting significantly greater disrespect and mistreatment compared to women with higher socioeconomic status (*M* = 48.55, *SD* = 19.73), *t*_(592)_ = 2.56, *p* = 0.011. Regional differences were also observed. Women who gave birth in southern Italy reported higher levels of disrespect and mistreatment, *F*_(2, 617)_ = 9.60, *p* < 0.001, partial η^2^ = 0.030. Specifically, those delivering in southern Italian regions (*M* = 55.73, *SD* = 23.25) experienced significantly more mistreatment (*p* < 0.001) compared to women in northern regions (*M* = 47.71, *SD* = 19.71). No significant differences were observed between scores of women giving birth in central Italian regions (*M* = 52.70, *SD* = 20.69) and those from other areas. Mothers of firstborn children (*M* = 52.81, *SD* = 21.76) reported significantly more disrespect and mistreatment during childbirth compared to mothers of laterborn children (*M* = 44.21, *SD* = 18.39), *t*_(618)_ = −4.06, *p* < 0.001. No significant differences were found based on child gender, *t*_(618)_ = −0.31, *p* = 0.75, or between mothers of full-term and preterm children, *t*_(618)_ = 1.92, *p* = 0.06.

Regarding childbirth-related variables, delivery type significantly influenced women's experiences of disrespect and mistreatment, as assessed by the DMCQ, *F*_(3, 616)_ = 32.59, *p* < 0.001, partial η^2^ = 0.14. Women who experienced an emergency cesarean section (*M* = 66.02, *SD* = 24.44) reported significantly higher DMCQ scores compared to those who had vaginal deliveries (*M* = 45.90, *SD* = 17.86; *p* < 0.001), scheduled cesareans (*M* = 51.87, *SD* = 18.86; *p* < 0.001) and instrumental deliveries (*M* = 59.19, *SD* = 25.43; *p* = 0.05). Women experiencing a scheduled cesarian cut (*p* = 0.04) and instrumental delivery (*p* < 0.001) reported significantly higher scores in the DMCQ than those having a spontaneous vaginal delivery. Labor duration also affected childbirth experiences, with women whose labor exceeded 12 h (*M* = 58.92, *SD* = 24.48) reporting more negative experiences than those with shorter labor (*M* = 46.80, *SD* = 18.61), *t*_(303.87)_ = −6.00, *p* < 0.001. Similarly, women who underwent episiotomy (*M* = 60.06, *SD* = 23.31) experienced more disrespect and mistreatment compared to those who did not, *t*_(132.09)_ = −4.45, *p* < 0.001. The administration of anesthesia during childbirth (*M* = 55.12, *SD* = 22.81) was also associated with higher DMCQ scores, *t*_(615.65)_ = −5.32, *p* < 0.001. Furthermore, childbirth complications (*M* = 67.08, *SD* = 24.58) were linked to greater disrespect and mistreatment, *t*_(69.42)_ = −5.50, *p* < 0.001, as were health issues in the newborn (*M* = 57.15, *SD* = 24.78), *t*_(167.92)_ = −3.40, *p* < 0.001.

### 3.3 Examining the associations between disrespect and mistreatment during childbirth questionnaire and parenting stress (aim 3)

The associations between women's negative childbirth experiences and parenting stress are illustrated in Table 6. Women who reported more disrespect and mistreatment during childbirth also reported higher stress perceived during parenting. The association with the parent-child dysfunctional interaction scale was not significant.

**Table 6:** Correlations between disrespect and mistreatment during childbirth questionnaire (DMCQ) and parenting stress index parental distress and parent-child dysfunctional interaction scores.

	***N***	***M***	***SD***	**1**	**2**
1. DMCQ Tot	620	51.07	21.39	–	
2. PSI PD	545	2.68	0.72	0.20^***^	–
3. PSI P-CDI	547	1.97	0.35	0.07	0.30^***^

DMCQ Tot, disrespect and mistreatment during childbirth total score; PSI PD, parenting stress index parental distress; PSI P-CDI, parent-child dysfunctional interaction. ^***^p < 0.001.

### 3.4 Identifying and characterizing women at high risk of experiencing disrespect and mistreatment during childbirth (aim 4)

A cut-off at the 90th percentile, corresponding to a score of 81, was used to identify women at high risk of experiencing disrespect and mistreatment during childbirth. Given the use of a 7-point Likert scale ranging from 1 (“Not at all”) to 7 (“Very much”), respondents with scores above 81 indicated that, on average, the questionnaire items reflected their personal experiences at least moderately. Women above this value were 67, the 10.8% of the sample, and were considered at high risk of perceiving disrespect and mistreatment during childbirth. Analysis showed that high-risk women reported more perinatal PTSD (*M* =3.35, *SD* =0.77) than women with scores below the 90° percentile (*M* =2.29, *SD* =0.73), *t*_(618)_ = −11.17, *p* < 0.001. Similar results were obtained for the subscale parental distress of the Parenting Stress Index, with women with DMCQ scores > 90° percentile (*M* = 2.86, *SD* = 0.64) showing higher scores than women scoring < 90° (*M* = 2.66, *SD* = 0.72), *t*_(618)_ = −2.12, *p* = 0.035. No differences were found among women above or below the cut-off for the Parent-Child Dysfunctional Interaction subscale of the Parenting Stress Index.

In terms of individual and contextual risk factors, women at high risk of experiencing significant disrespect and mistreatment during childbirth were more likely to have a high level of education and to have given birth in a southern Italian region. Being a first-time mother also emerged as a risk factor. Regarding childbirth-related variables, high-risk women were more likely to have experienced labor lasting longer than 12 h, undergone an emergency cesarean section, received an episiotomy, or used anesthesia during labor. Additionally, complications during childbirth and infant health issues were more prevalent in the high-risk group compared to the low-risk group. Detailed results are provided in Supplementary material s2 and s3.

## 4 Discussion

Disrespect and mistreatment during childbirth are widespread issues affecting both high-income and low-income countries. The World Health Organization has identified it as a global concern with significant social and health consequences that require urgent prevention (World Health Organization, 2014). However, research on this phenomenon is relatively recent, emerging primarily in the early 2000s, with pioneering studies originating in Latin America. Over the years, ongoing research and reflection have led to the development of various definitions, ultimately framing obstetric violence or mistreatment as a multidimensional phenomenon (Bohren et al., 2015; Darilek, 2018). This concept encompasses a range of disrespectful and abusive experiences faced by women during labor and childbirth.

Its complex definition is further compounded by the lack of appropriate tools to capture its dimensions fully. Much of the existing literature relies on *ad hoc* instruments that often fail to address all manifestations of disrespect and mistreatment women comprehensively may experience during childbirth. Moreover, these tools frequently lack psychometric validity, limiting their reliability and effectiveness. Most studies, for example, focus on the execution of medical procedures, which cannot always be equated with mistreatment, as these interventions can be crucial for ensuring a safe delivery and the health of the newborn. There is an urgent need to place greater emphasis on the psychological dimensions of mistreatment to better understand and address its impact on women's childbirth experiences.

This research aimed to advance the understanding of obstetric mistreatment in a high-income country like Italy by developing a comprehensive and psychometrically valid self-report questionnaire to capture women's experiences of disrespect and mistreatment during childbirth. To achieve this objective, we first conducted a thorough review of recent literature on obstetric mistreatment, including its prevalence, associated risk and protective factors, and its consequences for maternal mental health. A pool of 30 items was then administered to a sample of 620 women within 0–24 months postpartum. By focusing on this specific population, the study sought to explore how obstetric mistreatment impacts maternal mental health and parenting stress during the critical first year of parenting (Britto et al., 2017). The explorative factorial analysis led to a 20-item scale measuring the following five dimensions of disrespect and mistreatment during childbirth: negative interactions with healthcare providers, separation from the newborn, medical intrusiveness, verbal mistreatment, and pain experience. Further results showed that the DMCQ dimensionality yielded adequate fit indexes, and its subscales demonstrated good reliability.

### 4.1 Dimensionality and validity of the disrespect and mistreatment during childbirth questionnaire

The dimension labeled “Negative interaction with healthcare providers” accounted for the largest proportion of variance, underscoring its central role in obstetric mistreatment. This dimension highlights the critical importance of a woman's need to engage with responsive and empathetic healthcare providers during childbirth—a moment of profound vulnerability and stress. During labor, women rely on clear and effective communication with medical staff to access vital information about their baby's health and the progression of labor. Equally important is the assurance that the staff is available, attentive, and supportive, addressing their needs and providing care that fosters trust and understanding. Lack of support and communication from healthcare providers was identified as particularly significant in the study by van der Pijl et al. (2022) conducted in the Netherlands. Their findings revealed that 90% of the women who reported feeling ignored or being told they were overreacting found these behaviors especially distressing, compared to 70% who judged physical mistreatment as upsetting. Based on these results, van der Pijl et al. (2022) emphasized the importance of what they termed “subtle forms of disrespect”—such as imbalances in communication and control within the provider-patient relationship—and their role in shaping women's evaluation of their childbirth experience. Adequate communication with the healthcare staff constituted a protective factor for the development of maternal PTSD in the systematic revision of Silva-Fernandez et al. (2023) examining the factors associated with obstetric violence implicated in the development of women's mental health issues.

The second dimension emphasized by our findings pertains to the separation from the newborn. This includes the mother's sense of being abruptly separated from the baby and feeling insufficiently close to him/her in the immediate postpartum period. This aspect was included in Martínez-Galiano et al. (2021) and Martinez-Vázquez et al. (2022) studies in the cluster of items referred to as psycho-affective violence and also mentioned by Silva-Fernandez et al. (2023). According to the World Health Organization (1998), healthy full-term infants should be placed in skin-to-skin contact with their mothers immediately after birth. This practice is widely recognized as the optimal way for a newborn to adapt to life outside the womb, offering both short- and long-term benefits (Phillips, 2013). Research has shown that skin-to-skin contact supports the newborn's thermal regulation and significantly increases the likelihood of exclusive breastfeeding at the time of hospital discharge (Gabriel et al., 2010). Additionally, it has been demonstrated to reduce maternal postpartum depression and physiological stress during the first months after birth (Bigelow et al., 2012).

The third dimension, medical intrusiveness, reflects women's experiences of undergoing obstetric procedures without their consent or prior notification or perceiving certain procedures as excessively invasive. A lack of choice was identified as the most frequently reported form of disrespect and mistreatment in the study by van der Pijl et al. (2022). Similarly, Scandurra et al. (2022) found that approximately 56% of women participating in their study reported experiencing at least one instance of non-consented care. These included practices such as the use of electronic fetal monitoring despite a low-risk pregnancy or, more critically, the absence of consent for a cesarean section. However, as noted in the introduction, Scandurra et al. (2022) included women aged 18 to 60 in their study, suggesting that these practices might be less prevalent or potentially outdated for younger or more recent cohorts.

The medical intrusiveness dimension does not critique the use of obstetric procedures themselves but highlights the absence of adequate communication and a proper consent process. While certain interventions, such as episiotomies or specific birthing positions, may be necessary for the safe progression of delivery or emergencies—and are often accepted by women under these circumstances—their routine application without consultation or consent can lead to feelings of disrespect and mistreatment. For instance, episiotomies, as noted in the literature, have increasingly become standard practice, with women seldom being asked for their consent (Djanogly et al., 2022), exacerbating these negative experiences.

Verbal mistreatment, our fourth dimension, is a recurring aspect of obstetric mistreatment frequently highlighted in the literature (Bohren et al., 2015). In many Spanish studies on this topic (Martínez-Galiano et al., 2021; Martinez-Vázquez et al., 2021, 2022), verbal abuse—including verbal invalidation, inappropriate verbal treatment, and criticism of emotional expression—has been identified as one of the three core dimensions of obstetric violence, alongside physical and psycho-affective violence. Additionally, in Martínez-Galiano et al. (2021), verbal mistreatment was the most frequently reported form of violence experienced by women. The items included in the DMCQ verbal mistreatment factor regard both overt forms of verbal abuse (i.e., use of vulgar language and insults) and verbal invalidation (i.e., receiving demeaning remarks). The final dimension identified pertains to women's experiences of intense pain. In the qualitative study by Annborn and Finnbogadóttir (Annborn and Finnbogadóttir, 2022), inadequate pain relief emerged as a key theme in the content analysis of interviews. Women reported pain caused by the refusal of healthcare staff to administer anesthesia and by being forced to endure prolonged delays. During our analysis of the initial item pool, two items that could have captured these nuances were removed due to poor psychometric properties: “The healthcare staff delayed too long in providing me with pain relief” and “Even though I needed it, I was not provided with adequate pain relief.” This exclusion could have resulted in lower correlations between this dimension and other dimensions of the DMCQ and the poor amount of variance explained by this factor, issues that will be addressed further in the limitations section.

The convergent and divergent validity of the questionnaire were demonstrated. As anticipated, the total score of the DMCQ showed no association with the Big Five personality trait of openness, indicating that the two instruments measure distinct psychological constructs. The DMCQ total score did, however, correlate with the PPQ-II, which assesses perinatal posttraumatic symptoms. While obstetric mistreatment and perinatal PTSD are indeed separate constructs, the former—disrespect and mistreatment during childbirth—can serve as a causal factor in the development of posttraumatic symptoms, as well documented in the literature (Martinez-Vázquez et al., 2021; Silva-Fernandez et al., 2023).

### 4.2 Sociodemographic and childbirth-related variables associated with disrespect and mistreatment during childbirth

A further aim of the present study was to examine the sociodemographic and childbirth-related factors associated with more significant experiences of disrespect and mistreatment during childbirth. Maternal age did not emerge as a variable linked to differences in these experiences, consistent with findings by Ravaldi et al. (2018) and Martínez-Galiano et al. (2021). However, other studies have reported an effect of maternal age on obstetric mistreatment, showing that younger women are more likely to experience disrespect and mistreatment during childbirth (Scandurra et al., 2022; van der Pijl et al., 2022) Notably, this effect was observed primarily in women under 25 at the time of birth—a demographic that was underrepresented in our sample.

In line with Martínez-Galiano et al. (2021) and van der Pijl et al. (2022) but differing from others (Scandurra et al., 2022), women with higher educational levels in our study reported higher levels of disrespect and mistreatment. At the same time, however, women with lower monthly incomes were at greater risk of receiving mistreatment from healthcare providers. These findings together could seem controversial as typically low SES and low education were found to be associated with more significant forms of mistreatment. In recent years, spurred by the efforts of advocacy groups and NGOs in Europe, the media have increasingly covered the issue of obstetric mistreatment, likely contributing to greater awareness of the topic among women. Therefore, women with higher educational levels could be better equipped to recognize and identify acts of disrespect and mistreatment, be more aware of their rights, and recognize situations and acts in which they are not fully informed more easily. This can lead them to report more occurrences of disrespect and mistreatment.

Another interesting result showed that women giving birth in southern regions of Italy were at higher risk of receiving mistreatment during labor and delivery. This finding is consistent with Ravaldi et al. (2018), who reported higher obstetric violence in regions of the Center and South of Italy.

This is not surprising, given the significant variations in the quality of healthcare systems across Italy's regions. A well-documented and persistent North-South divide in hospital efficiency and performance has been consistently reported (Barra et al., 2022).

Primiparity also emerged as a factor associated with experiences of disrespect and mistreatment. Women delivering their firstborn were more likely to score higher on the DMCQ. The literature, however, presents inconsistent findings regarding this variable. While some studies report no significant effect of primiparity, others suggest greater distress among primiparous mothers, while still others highlight higher levels of mistreatment in multiparous women (Vedam et al., 2019; Scandurra et al., 2022; van der Pijl et al., 2022). Our interpretation of this finding is that primiparous mothers may be less prepared for the childbirth experience and possibly less informed about their rights and options. This lack of preparation could prompt healthcare providers to adopt a more assertive approach, potentially disregarding the mother's preferences and leading to reduced communication and informed consent.

The second set of variables examined focused on various aspects of childbirth, including obstetric procedures. As extensively documented in the literature (Martínez-Galiano et al., 2021; Scandurra et al., 2022), emergency cesarean deliveries were associated with the highest risk of obstetric mistreatment, followed by instrumental deliveries and scheduled cesarean births. In the study by Martínez-Galiano et al. (2021), instrumental birth and cesarean sections were specifically linked to higher levels of perceived physical and psycho-affective violence, but cesarean sections appeared to offer some protection against verbal violence. This finding was attributed to the nature of cesarean procedures, during which the active participation of the woman is not required, reducing the likelihood of verbal abuse from medical staff. In the Italian sample of Scandurra et al. (2022), women who received a cesarian section felt they were not appropriately informed about the procedure or did not consent to it. Other aspects of childbirth, such as the use of episiotomy, labor lasting more than 12 h, and complications during delivery for both mother and child, were found to be associated with experiences of disrespect and mistreatment. These factors often contribute to childbirth being perceived by women as particularly stressful and/or painful while also potentially impacting the mental state of medical providers (Grekin and O'Hara, 2014; Schrøder et al., 2016). Such high-stress contexts could create a fertile ground for the occurrence of obstetric mistreatment. Finally, the administration of anesthesia during childbirth was also associated with obstetric violence. This finding aligns with Martínez-Galiano et al. (2021), who reported a higher incidence of obstetric violence among women who received analgesia. The provision of anesthesia may involve elements perceived as mistreatment, such as prolonged waiting times or restrictions on mobility during labor. Furthermore, delayed anesthesia may reduce its effectiveness as labor progresses, leading to a more stressful experience.

### 4.3 Impact of disrespect and mistreatment during childbirth on parenting stress

Recent literature has shown that experiencing obstetric mistreatment is a significant risk factor for developing both posttraumatic stress symptoms—an association also evident in our findings—and postpartum depression (Martinez-Vázquez et al., 2021, 2022; Silva-Fernandez et al., 2023). However, to the best of our knowledge, research on how obstetric mistreatment may impact the mother-child relationship is still lacking, despite a strong theoretical basis suggesting this connection is likely to be significant. In our study, we looked at associations between experience of disrespect and mistreatment during childbirth and levels of parenting stress in mothers of children aged 0 to 2. We found that women showing higher levels of disrespect and mistreatment also reported greater distress in their parental role, whereas they did not report their interaction with their children to be particularly stressful. As noted in the introduction, childbirth is a pivotal experience for women, with profound psychological implications and both short- and long-term consequences (Stern and Bruschweiler-Stern, 1998). Obstetric mistreatment represents a profound wound, with potentially far-reaching effects on parenting and the parent-child relationship, underscoring the urgent need for increased attention and targeted interventions in this area.

### 4.4 Limits and suggestions for future studies

Despite its numerous strengths, this study presented some limitations that deserve attention.

First, the results of the exploratory factor analysis indicated fit indices that were adequate but not exceptional. This outcome may be attributed to the Pain Experience factor, which demonstrated adequate yet relatively low correlations with the total score and other subscales. Several items associated with this factor, particularly those addressing healthcare denial or delays in providing anesthesia, were removed due to their multiple loadings. While this adjustment may have improved the fit indices, it also resulted in a factor that lacks some important nuances. It would be interesting to explore whether this factor proves to be more functional in high-risk samples, such as situations involving highly medicalized childbirth and significant pain during and after delivery. Second, the use of the PPQ-II to assess convergent validity instead of tools specifically designed to measure obstetric mistreatment was not ideal. However, to our knowledge, this is the first Italian tool to measure disrespect and mistreatment during childbirth, thus filling an important gap in the literature. Scandurra et al. (2022) utilized an *ad hoc* questionnaire, while Ravaldi et al. (2018) adapted a Mexican tool for their study. Although Ravaldi et al. (2018) reported good reliability for the two factors included in the scale, neither of these instruments has undergone formal validation. Third, our sample, although appropriate in size, consisted almost only of Caucasian women, limiting the diversity captured. Fourth, the cross-sectional design of the study limited our ability to explore the long-term implications of experiences of disrespect and mistreatment during childbirth on maternal mental health and parenting. Longitudinal studies exploring the short- and long-term effects of disrespect and mistreatment during childbirth on maternal wellbeing and parenting could provide valuable insights in this regard.

### 4.5 Implications for clinical practice and research

This study has significant implications for both clinical practice and research, particularly concerning women's and children's wellbeing. Obstetric mistreatment is a widespread issue that transcends socioeconomic and cultural boundaries, with high prevalence rates reported in both low- and high-income countries. Despite growing awareness, previous research has often relied on *ad hoc* questionnaires and focused predominantly on the use of obstetric practices rather than on women's subjective experiences or perceptions of disrespect and mistreatment during childbirth.

Our study addresses this critical gap by developing a brief, comprehensive, and psychometrically validated self-report questionnaire specifically designed to measure obstetric disrespect and mistreatment. The availability of this instrument equips clinicians and researchers with a reliable tool to explore the clinical implications of such mistreatment, including its impact on maternal mental health (e.g., postpartum stress and depression) and its potential influence on the development of a healthy mother-child relationship. Additionally, the establishment of a clinical cut-off enhances the utility of this tool, enabling clinicians to identify women at higher risk of experiencing disrespect and mistreatment. This facilitates the development of targeted interventions, promoting better maternal and child health outcomes. Furthermore, this instrument provides a solid foundation for future research to investigate the prevalence, predictors, and long-term consequences of obstetric mistreatment on women and their families.

## Data Availability

The raw data supporting the conclusions of this article will be made available by the authors, without undue reservation.
