# Supplementary material for: Development and validation of the Disrespect and Mistreatment during Childbirth Questionnaire: risk factors and effects on parenting stress
Source: Front Psychol. 2025 Mar 5;16:1562679. doi: 10.3389/fpsyg.2025.1562679 (PMC11922090; doi:10.3389/fpsyg.2025.1562679)
Supplement: Supplementary file 1 [file Table_1.docx]

**Disrespect and Mistreatment in Childbirth Questionnaire – Italian version**

Table S1. *Italian Version of the Disrespect and Mistreatment during Childbirth Questionnaire*

| Original item number | Original item text | Factor |
| --- | --- | --- |
| 1 | Ho avuto la sensazione di essere sottoposta a manovre non necessarie | Medical intrusiveness |
| 2 | Ho avuto la sensazione di essere stata bruscamente separata dal mio bambino | Separation from newborn |
| 5 | *Gli operatori sono sempre stati disponibili* | Negative interaction with healthcare providers |
| 6 | Il personale medico-sanitario ha utilizzato un linguaggio volgare | Verbal mistreatment |
| 8 | Non so perché alcune procedure siano state effettuate | Medical intrusiveness |
| 10 | *Ho ricevuto il giusto supporto dal personale medico-sanitario* | Negative interaction with healthcare providers |
| 11 | Ho provato più dolore di quanto mi aspettassi | Pain experience |
| 12 | Sono stata insultata | Verbal mistreatment |
| 13 | *In caso di bisogno, sapevo sempre a chi rivolgermi* | Negative interaction with healthcare providers |
| 14 | Ho trovato eccessivamente invasive alcune procedure ostetriche a cui sono stata sottoposta | Medical intrusiveness |
| 16 | *Mio figlio è stato vicino a me quanto desideravo* | Separation from newborn |
| 17 | Durante il parto ho ricevuto frasi denigratorie | Verbal mistreatment |
| 19 | Ho ricevuto manovre ostetriche senza preavviso | Medical intrusiveness |
| 23 | In seguito al parto non sono stata vicino al mio bambino quanto avrei voluto | Separation from newborn |
| 24 | Ho temuto di non farcela per il troppo dolore | Pain experience |
| 25 | *Mi sono sentita in grado di affrontare l’esperienza* | Pain experience |
| 26 | Avrei desiderato un contatto maggiore col bambino appena nato | *Separation from newborn* |
| 27 | *Il personale medico-sanitario comprendeva i miei bisogni* | Negative interaction with healthcare providers |
| 28 | Non ho fornito un consenso per alcune procedure ricevute | Medical intrusiveness |
| 30 | Non ho potuto vedere il bambino per un periodo di tempo a parer mio eccessivo | Separation from newborn |

*Note*. Italicized Items should be reverse scored.

**Identifying and Characterizing Mothers at High-Risk of Disrespect and Mistreatment during Childbirth**

Table S2. *Independent T-Test of Risk Factors Between Low- and High-risk Mothers for Disrespect and Mistreatment during Childbirth*

|  | Low-risk group (*N* =67) | | High-risk group (*N* =553) | |  |
| --- | --- | --- | --- | --- | --- |
|  | *M* | *SD* | *M* | *SD* | *p* |
| Child Age | 14.46 | 5.01 | 13.53 | 5.01 | .15 |
| Maternal Age | 34.99 | 4.96 | 35.46 | 4.56 | .43 |

| Table S3. *Chi-squared Tests of Risk Factors Between Low- and High-risk Mothers for Disrespect and Mistreatment during Childbirth* | | | | | | | |
| --- | --- | --- | --- | --- | --- | --- | --- |
|  | Low-risk group (*N* =67) | | High-risk group (*N*=553) | |  |  |  |
|  | n Observed | n Expected | n Observed | n  Expected | χ^2^ | *df* | *p* |
|  |  |  |  |  |  |  |  |
| Maternal education |  |  |  |  | 4.00 | 1 | .046 |
| *Elementary/High school (1)* | **218** | **210.5** | **18** | **25.5** |  |  |  |
| *University/Postgraduate degree (2)* | **335** | **342.5** | **49** | **41.5** |  |  |  |
|  |  |  |  |  |  |  |  |
| SES |  |  |  |  | 2.77 | 1 | .096 |
| *< 2150€* | 192 | 198.1 | 30 | 23.9 |  |  |  |
| *>2150€* | 338 | 331.9 | 34 | 40.1 |  |  |  |
|  |  |  |  |  |  |  |  |
| Hospital area in Italy |  |  |  |  | 7.10 | 2 | .029 |
| *South* | **180** | **189.1** | **32** | **22.9** |  |  |  |
| *Centre* | 68 | 68.7 | 9 | 8.3 |  |  |  |
| *North* | **305** | **295.2** | **26** | **35.8** |  |  |  |
|  |  |  |  |  |  |  |  |
| Birth order |  |  |  |  |  |  |  |
| *Firstborn child* | **435** | **441.5** | **60** | **53.3** | 4.40 | 1 | .036 |
| *Laterborn child* | **118** | **111.5** | **7** | **13.5** |  |  |  |
|  |  |  |  |  |  |  |  |
| Birth type |  |  |  |  |  |  |  |
| *Preterm birth* | 46 | 48.2 | 8 | 5.8 | 0.99 | 1 | .321 |
| *Full-term birth* | 507 | 504.8 | 59 | 61.2 |  |  |  |
|  |  |  |  |  |  |  |  |
| Duration of labor |  |  |  |  | 26.24 | 1 | <.001 |
| *< 12 hours* | **348** | **330** | **23** | **41** |  |  |  |
| *> 12 hours* | **151** | **169** | **39** | **21** |  |  |  |
|  |  |  |  |  |  |  |  |
| Type of delivery |  |  |  |  | 36.31 | 3 | <.001 |
| *Natural delivery* | **380** | **361.2** | **25** | **43.8** |  |  |  |
| *Instrumental delivery* | 39 | 42.8 | 9 | 5.2 |  |  |  |
| *Cesarean section (scheduled)* | 51 | 49.9 | 5 | 6.1 |  |  |  |
| *Cesarean section (emergency)* | **83** | **99.0** | **28** | **12.0** |  |  |  |
|  |  |  |  |  |  |  |  |
| Episiotomy |  |  |  |  | 12.89 | 1 | <.001 |
| *No* | **462** | **451.8** | **44** | **54.2** |  |  |  |
| *Yes* | **80** | **90.2** | **21** | **10.8** |  |  |  |
|  |  |  |  |  |  |  |  |
| Anesthesia |  |  |  |  | 7.93 | 1 | .005 |
| *No* | **265** | **254.2** | **20** | **30.8** |  |  |  |
| *Yes* | **287** | **297.8** | **47** | **36.2** |  |  |  |
|  |  |  |  |  |  |  |  |
| Complications during childbirth |  |  |  |  | 19.77 | 1 | <.001 |
| *No* | **499** | **488.7** | **50** | **60.3** |  |  |  |
| *Yes* | **44** | **54.3** | **17** | **6.7** |  |  |  |
|  |  |  |  |  |  |  |  |
| Newborn's health complications |  |  |  |  | 9.54 | 1 | .002 |
| No | **409** | **399.8** | **37** | **46.2** |  |  |  |
| *Yes* | **101** | **110.2** | **22** | **12.8** |  |  |  |

*Note*. Comparisons with standardized residuals were greater than [2] are presented in bold.
